# Colonization of Mouse Spermatogonial Cells in Modified Soft Agar Culture System Utilizing Nanofibrous Scaffold: A New Approach

**DOI:** 10.31661/gmj.v8i0.1319

**Published:** 2019-05-09

**Authors:** Ali Talebi, Mohammad Ali Sadighi Gilani, Morteza Koruji, Jafar Ai, Mohammad Jafar Rezaie, Shadan Navid, Majid Salehi, Mehdi Abbasi

**Affiliations:** ^1^Department of Anatomy, School of Medicine, Tehran University of Medical Sciences, Tehran, Iran; ^2^Department of Urology, Shariati Hospital, Tehran University of Medical Sciences, Tehran, Iran; ^3^Cellular and Molecular Research Center & Department of Anatomical Sciences, Iran University of Medical Sciences, Tehran, Iran; ^4^Department of Tissue Engineering and Applied Cell Sciences, School of Advanced Technologies in Medicine, Tehran University of Medical Sciences, Tehran, Iran; ^5^Department of Embryology, Faculty of Medicine, Kurdistan University of Medical Sciences, Sanandaj, Iran; ^6^Department of Tissue Engineering, School of Medicine, Shahroud University of Medical Sciences, Shahroud, Iran

**Keywords:** Adult Germline Stem Cells, Cell Proliferation, Tissue Scaffolds, Agar

## Abstract

**Background:**

Spermatogonial stem cells (SSCs) are considered in fertility management approaches of prepubertal boys facing cancer therapies. However, in vitro propagation has become an important issue due to a small number of SSCs in testicular tissue. The present study aimed to investigate a modified soft agar culture system by using a nanofibrous scaffold as a new approach to mimic in vivo conditions of SSCs development.

**Materials and Methods:**

The SSCs were isolated from neonate mouse testes, cultured on polycaprolactone scaffold, and covered by a layer of soft agar for 2 weeks. Then, the number and diameter of colonies formed in experimental groups were measured and spermatogonial markers (i.e., *Plzf*, *Gfrα1*, *Id4*, and *c-Kit*) in SSCs colonies were evaluated by a real-time polymerase chain reaction and immunostaining.

**Results:**

Our results indicated that the colonization rate of SSCs was significantly higher in the present modified soft agar culture system (P<0.05). Only *Plzf* indicated a significant increased at the levels (P<0.05), the gene expression levels of *Id4*, *Plzf*, and *Gfrα1* were higher in the present culture system. In addition, the expression of the *c-Kit* gene as a differentiating spermatogonia marker was higher in presence of scaffold and soft agar compared with the amount of other experimental groups (P<0.05).

**Conclusion:**

The culture system by using nanofibrous scaffold and soft agar as a new culture method suggests the potential of this approach in SSCs enrichment and differentiation strategies for male infertility treatments, as well as in vitro spermatogenesis.

## Introduction

Although chemo- and radiotherapies play an important contribution to the cure of both oncological and benign diseases, they present the risk of fertility impairment. Recent estimates have shown that approximately one in 530 young adults between the ages of 20 and 39 is a childhood cancer survivor [1]. Sperm cryopreservation is regarded as the main option for preserving adult men fertility although it is not applicable in prepubertal boys who are not capable of producing sperm and their seminiferous tubules only contain spermatogonial stem cells (SSCs) [2]. In these cases, cryobanking testicular tissue or SSCs can be proposed for fertility preservation and should be done before starting gonadotoxic cancer therapies. Testicular tissue biopsies or SSCs suspensions may undergo transplantation processes or in vitro spermatogenesis for fertility restoration [2, 3]. In addition, the use of SSCs has created some challenges, along with complications to achieve successful transplantation of testicular tissues. Further, in vitro spermatogenesis through safe protocols has been considered as a clinical approach in these cases since auto-transplantation can be related to the risk of contaminating malignant cells [4]. SSCs, as adult germline stem cells, are located in seminiferous tubules in testis and are responsible for continuous spermatogenesis during adult male life. SSCs are a small subpopulation of type A spermatogonia with only about 0.02%–0.03% of all the cells in the mammalian testicular tissue, where they can self-renew to maintain the stem cell pool and diﬀerentiate to produce mature spermatozoa [5]. Therefore, SSCs transplantation techniques need a sufficient amount of SSCs for colonizing recipient seminiferous tubules. There are several strategies for isolating and purifying for the purpose of separating a pure population of SSCs from testicular tissue [5, 6]. SSCs can be propagated artificially through different in vitro culture systems which should maintain their survival, proliferation, and stemness. Long-term in vitro culture of SSCs was reported in several species such as rodents and humans [7]. Further, culture systems should ideally simulate the *in vivo* condition as similar as possible although in vitro mimicking of the SSC niche environment is very difficult. Electrospun nanofiber scaffolds are regarded as artificial matrices which display morphological similarities to the extracellular matrix (ECM) such as basement membrane and are extensively used in tissue engineering [8]. In order to tissue scaffold fabrication, natural and synthetic polymers can be applied. Polycaprolactone (PCL) is a biocompatible and biodegradable synthetic polymer which is incorporated in different formulations for tissue engineering applications [9].Spermatogenesis is orchestrated by different factors and somatic cells such as Sertoli, Leydig and myoid cells in 3-dimensional (3D) architecture of seminiferous epithelium [10]. Various 3D culture systems and ECM proteins have been emphasized for proliferation and differentiation of SSCs due to the significance of 3D microenvironment and since structural conditions, which may closely resemble the in vivo testicular microenvironment, are not provided in 2D conventional culture approaches. Stukenborg *et al*. introduced a soft agar culture system (SACS) for propagating and differentiating mouse SSCs and accordingly the differentiation of murine SSCs to post-meiotic stages and spermatozoa were reported [11, 12]. Although they demonstrated positive effects of soft agar on the propagation and differentiation of SSCs, they failed to isolate live spermatozoa from their culture systems [12]. In order to overcome this limitation in SACS, combined effects of PCL/gelatin (PCL/Gel) nanofibrous scaffold and soft agar was evaluated as a new culture system on proliferating neonate mouse SSCs in the present study.

## Materials and Methods

### Animal

Testicular cells were obtained from 3–6 day-old Naval Medical Research Institute (NMRI) male mice. Ten neonate mice were used for each repeat in all experimental groups. Then, animals were housed and bred under controlled conditions (12:12 light-dark cycle and 22-25oC temperature) and were provided with water and standard laboratory chow (Laboratory animals feed, Javane Khorasan Co, Tehran, Iran). In the next stage, all animal care and experimental procedures were performed according to the Ethics Committee of Tehran University of Medical Sciences (approval code: IR.TUMS.MEDICINE.REC.1396.2507).

### Spermatogonial Cell Isolation

Neonate mouse testes were collected and testicular cells were isolated by two-step enzymatic method after removing tunica albuginea. The digestion solution included hyaluronidase (Sigma-Aldrich Corp., St. Louis, MO, USA, 0.5 mg/mL), collagenase type IV (Gibco, Life Technologies corp., USA, 1 mg/mL), and DNase (Sigma-Aldrich Corp., St. Louis, MO, USA, 10 μg/mL). Then, testicular tissue fragments were suspended into the digestion solution for 20 min at 37°C in a 5% CO2 incubator and centrifuged at 1,500 g for 5 min. Then, fresh digestion solution was added to the cell pellet and incubated for more 15 min. In every digestion step, pipetting was done for every 5 min. Testicular cells were then centrifuged at 1,500 g for 5 min. SSCs were purified by using differential plating method and the same modifications described in the previous study [13].

### Fabrication of Scaffold

PCL (Sigma-Aldrich Corp., St. Louis, MO, USA, 14% w/v) and Gel (Sigma-Aldrich Corp., St. Louis, MO, USA, 14% w/v) solutions were 1:1 mixed and stirred for 24 hours. The syringe containing polymer solution was placed in a syringe pump (Fanavaran Nano-Meghyas, Tehran, Iran) connected to a collector with a 15-cm distance from the syringe needle tip. The feed rate of the pump was kept at 0.4 mL/h and the voltage was maintained at 20 kV by a high voltage supply (Fanavaran Nano-Meghyas, Tehran, Iran). In addition, scanning electron microscope (SEM, CamScan, MV2300, UK) was used in examining the scaffold morphology and fiber diameter was measured from SEM images by using the image analysis software ImageJ (National Institutes of Health, USA). Total porosity calculation was done by liquid displacement method using ethanol [14]. The scaffold wettability was tested by the Sessile drop method by using a contact angle measuring system (KRUS, Hamburg, Germany) [15].

### Soft Agar Culture Using Nanofibrous Scaffold

Four experimental groups including the control group: conventional culture, experiment (Exp.) 1: conventional culture system using soft agar, Exp. 2: PCL scaffold, and Exp. 3: PCL scaffold in soft agar. In all groups, spermatogonial cells were seeded at 2×105 cells/well in a 24-well culture plate. In Exp. 1 and 3, the culture medium was replaced by 0.03% agar and kept in an incubator at 37°C with 5% CO2 for 2 weeks after 4 days of completing cell adhesion to scaffold and petri dish. Culture medium was Minimum Essential Medium Eagle solution (alpha-MEM, Sigma-Aldrich Corp., St. Louis, MO, USA) which was supplemented with 1x nonessential amino acids (Invitrogen, USA), 10% fetal bovine serum (FBS, Sigma-Aldrich Corp., St. Louis, MO, USA), 100 U/mL penicillin (Sigma-Aldrich Corp., St. Louis, MO, USA), 100 μg/mL streptomycin (Sigma-Aldrich Corp., St. Louis, MO, USA), 0.1 mM 2-mercaptoethanol (Sigma-Aldrich Corp., St. Louis, MO, USA), 103 U/mL human recombinant leukemia inhibitory factor (LIF, Sigma-Aldrich Corp., St. Louis, MO, USA) and 10 ng/mL glial cell line-derived neurotrophic factor (GDNF, Sigma-Aldrich Corp., St. Louis, MO, USA).

### Real-Time Polymerase Chain Reaction (PCR)

After 2 weeks of culture, the expression of the spermatogonial markers, inhibitor of DNA binding protein-4 (*Id4*), promyelocytic leukemia zinc finger protein (*Plzf)*, GDNF family receptor alpha-1 (*Gfrα1*), as well as differentiation marker tyrosine-protein kinase Kit (*c-Kit*) were evaluated by real-time PCR (polymerase chain reaction). The total RNA was extracted by Trizol (Ready Mini Kit, Qiagen, USA) and cDNA synthesis was performed by manufactured kit (Transcript First Strand cDNA Synt, Roche, USA) in accordance with the manufacturer’s instructions. Real-time PCR was performed by using Applied Bioscience 7500 fast and SYBR Green. Each PCR reaction mixture containing 10 μl of 2X SYBR GreenMaster Mix, 0.5 μl of forward primer, 0.5 μl of reverse primer, and 1 μl of cDNA in a total volume of 20 μl. The cycling parameters included initial incubation at 95°C for 15 min, 40 cycles of 95°C for 10 s, 61°C for 15 s (59°C for *c-kit* and 58°C for *Gfrα1*), and 72°C for 10 s, with a final melting at 95°C for 10 s, 65 for 60 s and 97 for 60 s. For each sample, real-time PCR reactions were performed in triplicate. Melting curves were analyzed after each run and the samples were normalized against *β-actin* (internal control). Primer sequences are presented in Table-1.

### Immunostaining

Immunocytochemistry was performed for *Plzf* expression on SSC colonies. After 14 days, colonies were fixed with 4% paraformaldehyde and permeabilized with 2% Triton X-100, and then incubated in 1/100 diluted *Plzf* antibody for 2 h at 37 °C. After washing, 1/100 diluted Donkey Anti-Rabbit labeled with fluorescent isothiocyanate (FITC) (Sigma-Aldrich Corp., St. Louis, MO, USA) as a secondary antibody was added for 3 h at room temperature. Nuclei were counterstained with 4, 6-diamidino-2-phenylindole (DAPI, Sigma-Aldrich Corp., St. Louis, MO, USA, 1μg/mL).

### Statistical Analysis

All data were expressed as the mean ± standard deviation (SD). Statistical analysis of the results was performed by using repeated measure ANOVA followed by *Turkey post hoc* test in GraphPad Prism 7.0 (GraphPad Software, Inc. USA). A P-value< 0.05 was considered as statistically significant.

## Results

### Characterization of Scaffold

SEM imaging exhibited the non-woven porous structure of the scaffold with fibrils, which were oriented in a random manner. Table-2 indicates fiber diameter, porosity, contact angle, and the tensile strength of the scaffold.

### Colony Assay

**Figure 1:**
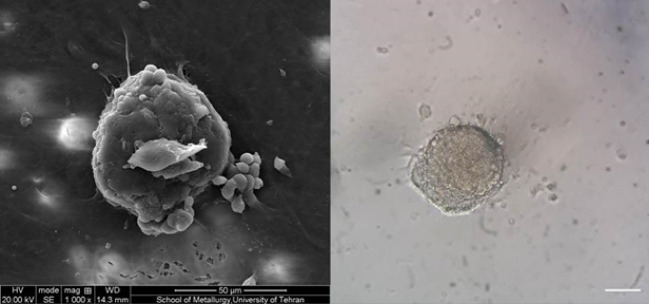
A: SEM image of SSCs colony formed in our modified culture system; B: inverted microscope image of SSCs colony formed in conventional culture system (scale bar: 50µm)

**Figure 2:**
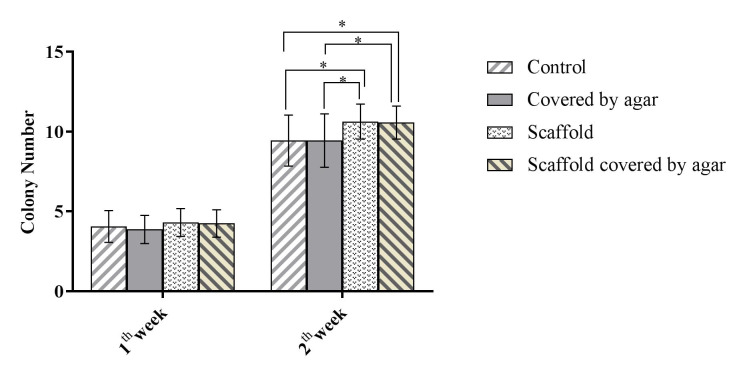
Number of SSCs colonies in designed experimental groups, * P<0.05.

**Figure 3:**
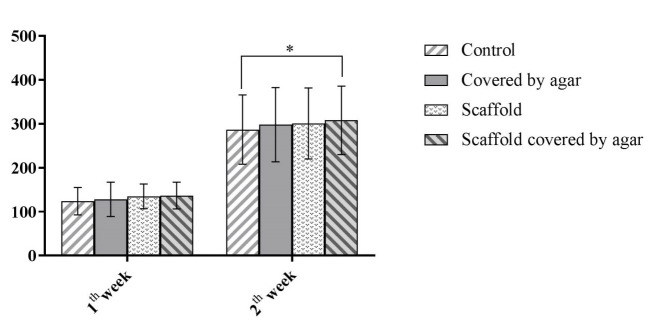
Diameter of SSCs colonies in designed experimental groups, * P<0.05

Figure-1 illustrates the SEM image of a colony in culture system and a bright field image of a colony in the conventional culture of SSCs. In addition, 4.06±0.9, 3.87±0.9, 4.31±0.8, and 4.25±0.8 colonies in control, Exp. 1, Exp. 2, and Exp. 3, respectively, at end of the first week (Figure-2) with no significant difference(P>0.05). At the end of the second week of cultivation, the mean number of colonies was significantly higher in the Exp. 2 and 3, compared to that of control and Exp. 1 (P<0.05). The mean number of colonies was calculated as 9.43±1.6, 9.43±1.7,10.62±1.1, and 10.56±1.0 in the control group, Exp. 1, Exp. 2, and Exp. 3, respectively. In addition, the mean diameter of colonies was 124±31.4 in control group, 128±39.2 in Exp. 1, 135±28.2 in Exp. 2 and 137±30.4 in Exp. 3 at the end of the first week of the culture period. At the end of the culture period, the mean diameter of colonies increased 287±79, 298±84.6 in, 301±81 in and 308±78 in, in control, Exp. 1, Exp. 2, and Exp. 3 group. As displayed in Figure-3, the mean diameter of colonies in Exp. 3 was significantly higher than that of other groups (P<0.05).

### Gene Expression

**Figure 4:**
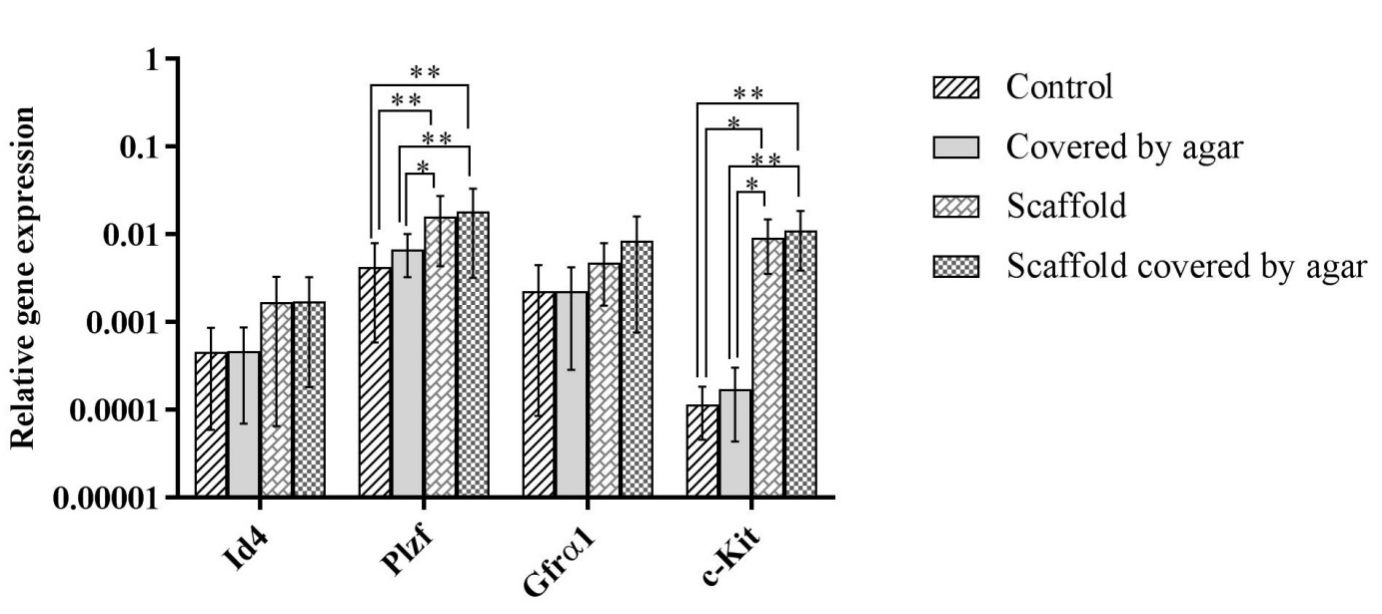
Expression of gene markers of spermatogonial cells in designed experimental groups, *P<0.05, ** P<0.01.

Results of real-time PCR were calculated by 2-Δct formula and normalized against housekeeping gene. Mean expression of *Id4* genes were 0.000461±0.0004, 0.000469±0.0004, 0.001683±0.0016, and 0.001731±0.0015 in control, Exp. 1, in Exp. 2, and Exp. 3 group, respectively. In addition, the expression of *Plzf* gene was 0.004279±0.0036 in control group, 0.006719±0.0034 in Exp. 1, 0.015921±0.0115 in Exp. 2 and 0.018259±0.0150 in Exp. 3. Further, gene expression of *Gfrα1* was 0.002277±0.0021, 0.002261±0.0019, 0.004779±0.0032, and 0.008424±0.0076 in control, Exp.1, Exp. 2, and Exp. 3 group. Finally, the expression of the *c-Kit* gene was 0.000115±0.00007 in control group, 0.000174±0.0001 in Exp. 1, 0.009224±0.0056 in Exp. 2 and 0.011194±0.0073 in Exp. 3. Figure-4 illustrates a graph of gene expressions.

### Immunostaining

**Figure 5:**
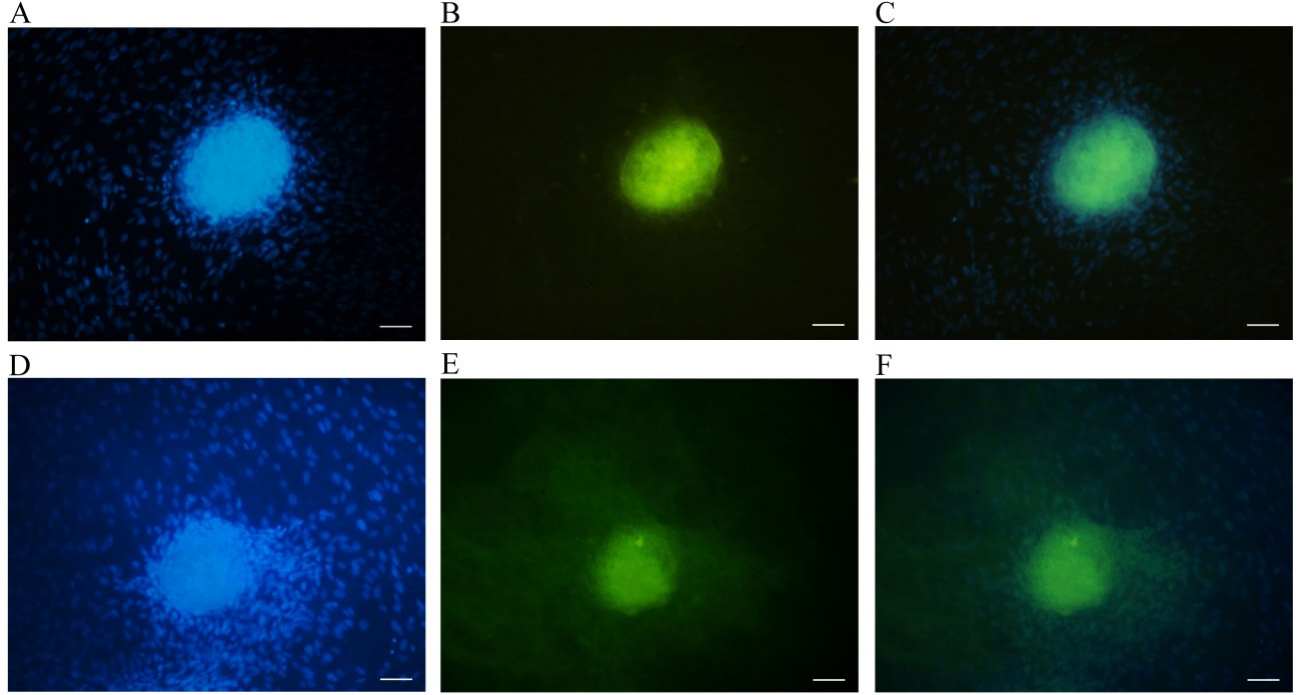
Immunostaining of PLZF for confirmation of spermatogonial colony identity in control group (upper row) and modified soft agar culture system utilizing PCL scaffold. Colonies were counterstained with DAPI (A and D), C and F are merged images (scale bar: 50µm).

In the present study, immunostaining was used as a qualitative assay for confirming SSCs colony identity and showing the expression of spermatogonial cell markers in colonies. Based on the results, the spermatogonial colonies were positive for *Plzf* immunostaining in all groups. As shown in Figure-5, a colony in the control group and a colony on the PCL scaffold was observed in the present culture system.

## Discussion

In the present study, mSSCs can be propagated and form colonies in a modified soft agar culture system by utilizing a nanofibrous scaffold. In the present approach, mSSCs had a higher proliferation rate rather than 2D conventional culture system. Thus, it seems likely that it can be employed in SSC enrichment protocols. The present developed culture system provides a tool for more effective strategies in SSC propagation, which are used in male infertility treatment and *in vitro* spermatogenesis. Most applications of SSCs in infertility management are related to prepubertal boys facing cancer and gonadotoxin therapies who are likely sterilized in their future life [16]. Among these patients, testicular tissue fragments should be harvested before the beginning of treatment and immature germ cells can be utilized in the approaches like in vitro spermatogenesis, autotransplantation and xenografting [3]. A sufficient amount of germ cells is needed for autotransplantation and *in vitro* spermatogenesis approaches and these cells should be propagated in vitro via various culture methods because of a small number of SSCs in testicular tissues. In the present study, a culture method was introduced by using soft agar and nanofibrous scaffold, which can support the SSCs propagation. In the present study, the improvement of mSSCs colonization was observed on the PCL/Gel scaffold and the scaffold in soft agar during a culture period. Therefore, it is concluded that mimicking ECM properties by nanofibrous scaffold can help cell attachment and proliferation due to more proliferation. The results were in line with the study of Karbalaei Mahdi et.al, which demonstrated that human induced pluripotent stem cells were more viable on PCL/Gel scaffold during culture for differentiation toward neuron [17]. In the case of providing a reliable structure with cell attachment sites by a composite of PCL as a synthetic polymer and gelatin as a natural polymer, several studies have been reported regarding the usefulness of these scaffolds in neural and skin regeneration [18, 19]. Based on the findings of different studies, nanofibrous surfaces can provide a more suitable microenvironment for proliferating and colonizing SSCs in vitro [20-22]. Shakeri *et al*. indicated the positive influence of nanofibrillar surface on the number of SSCs colonies in the culture system, leading to better expansion and function of SSCs due to the significance of topographic cues provided by nanofibrillar surfaces [22]. In the present study, the mSSCs seeded on the scaffold covered by an upper layer of soft agar could form more and larger colonies reflecting more proliferation of SSCs. Through mimicking topographic features of ECM, nanofibrous scaffolds are regarded as a favorable approach for improving the cell behaviors such as function, proliferation, and differentiation. Nowadays, 3D culture methods of SSCs are used to mimic the microenvironment of the in situ seminiferous epithelium. These systems can increase our understanding of the interactions between SSCs and somatic cells, as well as ECM which are involved in the process of male gametogenesis. Although several studies have implemented SACS in SSCs cultivation, more attention is needed to the differentiation [11, 12]. In the present study, the positive effects of nanofibrous surfaces on SSCs proliferation in SACS were highlighted. Since SACS is developed for SSCs differentiation, there is a problem to isolate live spermatozoa from the culture system [12]. Elhija *et al*. used SACS as two layers of different agar concentrations, meaning a lower layer as solid and an upper layer as gel. Although they could differentiate SSCs toward post-meiotic spermatozoa, they were able to identify sperms only after fixation process [12]. In the present modified culture system, we were able to remove soft agar and get access to the cells via trypsinization due to cell attachment to the scaffold. To date, SSCs have not been regarded as a unique marker for identification, the present study explored several marker gene expressions in colonies for determining their identity. Colonies were expressed as *Plzf*, *Id4,* and *Gfra1* as spermatogonial cell markers which revealed their undifferentiating status. Evidently, as SSC colonies can have differentiating spermatogonial cells, the expression of the c-Kit gene was evaluated as a differentiating marker in colonies simultaneously. In all experimental groups, SSC colonies indicated *c-Kit* gene expression with higher levels in grown colonies on PCL scaffold and the present modified culture system. Although the PCL scaffold could promote differentiation in SSCs, better results were obtained from more proliferation and larger colonies on the scaffold involving the number of differentiating spermatogonial cells. The results were in line with the study of Eslahi *et al*, which showed positive effects of poly-L-lactic acid usage in SSC culture system and suggested poly-L-lactic acid nanofibrous scaffolds as a useful method in supporting cell proliferation process [21]. However, SSCs should be proliferated in an undifferentiating status and their stemness properties should be maintained during in vitro propagation. Finally, the expression of differentiating genes such as *c-Kit*, as well as in the present approach, is considered as an unexpected result which can limit the strategies in SSCs enrichment.

## Conclusion

Based on the results, nanofibrous scaffold and soft agar can be used together in a new culture system for enriching mouse SSCs with higher efficiency than that of the conventional culture system. The present modified soft agar culture system as a new strategy in SSCs propagation can be utilized in male infertility management, especially in prepubertal boys suffering from cancer and in vitro spermatogenesis.

## Conflict of Interest

The authors declare that there are no conflicts of interest.

## Acknowledgment

We are grateful for the funding support provided by Tehran University of Medical Sciences (Grant No. 34398). The results described in this article were a part of a thesis.

**Table 1:** Primer Sequences Used for Real-Time PCR.

**Gene name**	**Primers (5’ to 3’)**
*Id4*	F: TCCCGCCCAACAAGAAAGTC R: TCAGCAAAGCAGGGTGAGTC
*Plzf*	F: CGTTGGGGGTCAGCTAGAAAG R: CACCATGATGACCACATCGC
*Gfra1*	F: AATTGTCTGCGTATCTACTGG R: ACATCTGATATGAACGGGAC
*c-Kit*	F: AACAACAAAGAGCAAATCCAGG R: GGAAGTTGCGTCGGGTCTAT
*β-actin*	F: TGTCCACCTTCCAGCAGATGT R: AGCTCAGTAACAGTCCGCCTAG

**Id4:** inhibitor of DNA binding protein-4; **Plzf:** promyelocytic leukemia zinc finger; **Gfrα1:** GDNF family receptor alpha-1; **c-Kit:** tyrosine-protein kinase Kit

**Table 2:** Characterization of PCL Scaffold. Data Are Presented as Mean±SD.

**Fiber diameter (nm)**	**Tensile strength (MPa)**	**Porosity (%)**	**Contact angel (** ^0^ **)**
692±72	3.01±0.74	70.1±5.3	76.7±0.9
